# Comparative effectiveness of remineralization agents on attachment-associated enamel demineralization in clear aligner patients: A 6-Month DIAGNOdent-Based controlled clinical trial

**DOI:** 10.1186/s12903-025-07497-8

**Published:** 2025-12-11

**Authors:** Hasan Yasin Ünal, Banu Kılıç, Evrim Eliguzeloglu Dalkilic

**Affiliations:** 1 Department of Orthodontics, Institute of Health Sciences, Bezmialem Vakif University, Fatih, Istanbul 34093 Turkey; 2 Department of Orthodontics, Faculty of Dentistry, Bezmialem Vakif University, Fatih, Istanbul 34093 Turkey; 3 Department of Restorative Dentistry, Faculty of Dentistry, Bezmialem Vakif University, Fatih, Istanbul 34093 Turkey

**Keywords:** Clear aligners, Orthodontic attachments, White spot lesions, DIAGNOdent, Nano-hydroxyapatite

## Abstract

**Background/objectives:**

Clear aligner attachments increase the risk of white spot lesions (WSL), with a 35.5% incidence in adolescents. The number of anterior attachments is an independent risk factor (OR = 2.192). Despite 17 million patients treated worldwide, no study has quantitatively assessed demineralization around attachment margins. To compare the effectiveness of CPP-ACP, nano-hydroxyapatite (nHAp), and fluoride varnish versus a control for attachment-associated demineralization using DIAGNOdent monitoring.

**Methods:**

This prospective controlled clinical trial evaluated 52 patients; 45 were enrolled, and 40 completed after five pre-baseline withdrawals. Participants were allocated to four groups (*n* = 10 each): Group A (control, fluoride toothpaste), Group B (CPP-ACP daily), Group C (nHAp professional + home gel), and Group D (fluoride varnish quarterly). DIAGNOdent measurements were taken around attachments at baseline, 1, 3, and 6 months. Linear mixed-effects models analyzed group differences (*p* < 0.05).

**Results:**

All 40 participants completed the 6-month study (100% retention). Baseline values were comparable (*p* = 0.48). Mean DIAGNOdent changes at 6 months were: Control + 4.35 [95%CI: 3.77–4.93], CPP-ACP − 4.02 [− 4.47 to − 3.56], nHAp − 5.36 [− 5.99 to − 4.72], Fluoride − 4.45 [− 4.78 to − 4.11]. The main group effect was significant (*p* < 0.001; η²*p* = 0.974). All treatments outperformed the control (*p* < 0.001). nHAp showed the greatest reduction, superior to CPP-ACP (*p* < 0.001) and fluoride (*p* = 0.019). Fluoride exhibited a biphasic pattern.

**Conclusions:**

All remineralization protocols significantly reduced demineralization versus control, with nHAp demonstrating the highest efficacy. Early preventive intervention and regular monitoring are recommended in attachment-bearing patients. Larger randomized trials are warranted.

**Trial registration:**

ClinicalTrials.gov NCT07229105. Registered 13 November 2025. Retrospectively registered.

**Supplementary Information:**

The online version contains supplementary material available at 10.1186/s12903-025-07497-8.

## Introduction

White spot lesions (WSLs) are the most prevalent iatrogenic complication of orthodontic treatment, affecting 2–97% of patients, depending on the assessment method and population studied [1, 2]. These incipient carious lesions, characterized by subsurface enamel demineralization with an intact surface layer [3], can develop within four weeks of plaque accumulation and progress to cavitation if left untreated [4]. Recent evidence from Liu et al. reveals a striking 35.5% WSL incidence in clear aligner-treated adolescents, with the number of anterior attachments emerging as an independent risk factor (OR = 2.192, 95% CI: 1.158–3.782) [5].

Clear aligner therapy, utilized in over 17 million patients globally, employs composite resin attachments to facilitate complex movements [6]. These attachments create plaque-retentive areas at the enamel-composite interface, where conventional hygiene measures prove insufficient [7, 8]. The attachment-enamel margin presents unique challenges, such as altered surface topography, potential microleakage, and disrupted salivary clearance patterns [9]. Despite the widespread use of attachments, no clinical studies have specifically monitored demineralization at high-risk sites.

The relationship between clear aligners and WSL development remains poorly understood, despite growing clinical concerns. Systematic reviews have revealed conflicting evidence, with one noting that “clear aligners were associated with low WSL risk, but there is limited evidence of a protective effect” compared to fixed appliances [2, 10]. A recent scoping review found that “among five included studies, only one reported WSLs and showed no significant difference versus fixed appliances” underscoring the paucity of high-quality evidence [8]. The economic implications are substantial, with treatment and retreatment of WSLs potentially costing up to €2,332 per patient [11], contributing to the global dental disease burden of US$298 billion annually [12].

Alternative remineralization technologies have emerged to address the limitations of fluoride monotherapies. Calcium-phosphate technologies, particularly casein phosphopeptide-amorphous calcium phosphate (CPP-ACP), have been studied extensively. While some policy reviews note protocol-dependent effectiveness, a comprehensive 2025 systematic review confirmed that CPP-ACP demonstrates a statistically significant effect on the prevention and regression of white spot lesions compared to placebo or standard care [13]. Nanohydroxyapatite (nHAp) has emerged as a key biomimetic agent. A 2024 systematic review focusing on hydroxyapatite products confirmed their promising and comparable efficacy in enamel remineralization [14]. This aligns with a randomized controlled trial showing that “the nHAp group had higher remineralization ability than CPP-ACP at 1 month using DIAGNOdent measurements“ [15].

Given the established risk of attachment-associated demineralization and the lack of evidence-based prevention protocols, this study aimed to compare the effectiveness of three remineralization strategies—CPP-ACP, nano-hydroxyapatite, and professional fluoride varnish—versus standard care in preventing enamel demineralization around clear aligner attachments using quantitative DIAGNOdent monitoring over six months. We hypothesized that there would be no significant difference (null hypothesis, H₀) in attachment-associated demineralization changes among CPP-ACP, nano-hydroxyapatite, fluoride varnish, and control groups as measured by DIAGNOdent over a 6-month period.

## Materials and methods

### Study design and setting

This prospective controlled clinical trial was conducted at Bezmialem Vakif University Dentistry Faculty Orthodontic Clinic from August 2024 to March 2025. The study protocol was approved by the Institutional Review Board (Protocol No: 09/15, date: 22/05/2024). All participants provided written informed consent, and parental consent was obtained from patients aged 16–18 years. This study followed the STROBE guidelines [16], adapted for the reporting of controlled clinical trials.

### Sample size and recruitment

This was an exploratory study using a convenience sample. No formal a priori sample-size calculation was performed. A total of 52 patients currently undergoing clear aligner therapy were assessed for eligibility between August and September 2024. Seven patients were excluded: two with systemic diseases, two who started medications affecting salivary flow, and three who declined participation. The remaining 45 patients provided written informed consent and were allocated to the four treatment groups.

Before the baseline measurements, five patients withdrew from the study. In Group B (CPP-ACP), two patients withdrew: one due to intolerance to sweet products with nausea during trial application and another due to concerns about potential allergic reactions given the dairy-based composition. In Group C (nHAp), one patient withdrew due to family relocation. In Group D (fluoride varnish), 2 patients withdrew their inability to attend regular clinical appointments. No withdrawals occurred in Group A (control). This resulted in 40 patients (*n* = 10 per group) undergoing baseline DIAGNOdent measurements, all completed the 6-month protocol (100% post-baseline retention).

### Eligibility criteria

#### Inclusion criteria

- Age 16–45 years.
- Active clear aligner treatment with minimum 6 months remaining.
- Presence of ≥ 10 attachments on anterior teeth and premolars.
- Good general health.
- Commitment to prescribed aligner wear (20–22 h/day).

#### Exclusion criteria

- Active carious lesions.
- Periodontal disease.
- Fluorosis or enamel hypoplasia.
- Current fluoride supplement use beyond standard toothpaste.
- Pregnancy or lactation.
- Systemic conditions affecting salivary flow.

### Group allocation and interventions

#### Group A: Control (*n* = 10)

The participants used standard fluoride toothpaste (1450 ppm) twice daily, with no additional remineralization protocol.

#### Group B: casein phosphopeptide-amorphous calcium phosphate (*n* = 10)

In addition to the standard fluoride toothpaste, participants applied GC Tooth Mousse (GC Corporation, Tokyo, Japan) to their clear aligners once daily. The protocol involved:

- Dispensing a pea-sized amount of mousse into each aligner.
- Wearing the aligner with mousse for 15 min.
- No rinsing for 30 min post-application.
- Application performed before bedtime.

#### Group C: nano-hydroxyapatite (*n* = 10)

This group received a combined and intensive protocol designed to maximize nano-hydroxyapatite exposure. It involved in-office applications of a nano-hydroxyapatite mineral gel (5% nHAp; BioWhiten ProOffice NanoCare Nano-Hydroxyapatite Mineral Jel, BiODENT MEDICAL, Istanbul, Turkey) performed by the examiner at baseline (T0), 1 month (T1), and 3 months (T2). This professional application was complemented by an at-home regimen consisting of daily application of a different nHAp dental care gel (1% nHAp; Biowhiten Nanocare nHAp Diş Bakım Jeli, BiODENT MEDICAL, Istanbul, Turkey), which participants squeezed into their aligners each night before sleeping (or for a minimum of 15 min if applied during the day). For routine daily brushing, participants in all four groups, including Group C, were provided with and instructed to exclusively use a standard fluoride toothpaste containing 1450 ppm fluoride.

#### Group D: professional fluoride varnish (*n* = 10)

The participants maintained the standard fluoride toothpaste use plus.

- Professional application of 5% sodium fluoride varnish (ProShield varnish, PRESIDENT DENTAL GmbH, Allershausen, Germany) at baseline and 3 months.
- Varnish applied to all tooth surfaces, with special attention to attachment margins.

− 1 min contact time before removal [17].

- No eating/drinking for 2 h post-application.

### Standardized oral hygiene protocol

For routine daily brushing (e.g., twice daily), participants in all four groups, including Group C, were provided with and instructed to exclusively use a standard fluoride toothpaste containing 1450 ppm fluoride. No other remineralizing toothpaste was permitted during the study period.

### DIAGNOdent measurement protocol

#### Calibration and standardization

The DIAGNOdent pen (KaVo, Biberach, Germany) was calibrated before each measurement session according to the manufacturer’s instructions using a ceramic standard [18]. A single calibrated examiner performed all measurements to ensure consistency. Intra-examiner reliability was assessed using intraclass correlation coefficient (ICC) with two-way mixed model, absolute agreement, calculated from 30 repeated measurements at 10 randomly selected attachment sites (ICC = 0.85, 95% CI: 0.79–0.91).

#### Measurement technique

A standardized measurement protocol was employed for all DIAGNOdent assessments. Teeth were air-dried for 5 s prior to measurement, and the probe tip was positioned perpendicular to the enamel surface to ensure consistent readings. Four measurements were obtained per attachment site at the mesial, distal, gingival, and occlusal margins. Three measurement passes were performed at each site, with the maximum value recorded to represent the most advanced demineralization present. Measurements were conducted at four timepoints: baseline (T0), 1 month (T1), 3 months (T2), and 6 months (T3).

#### Cut-off values

DIAGNOdent measurements were categorized using validated cut-off values for smooth enamel surfaces. Values between 0 and 12 were classified as sound enamel, scores of 13–24 indicated initial demineralization, and readings ≥ 25 represented advanced demineralization [18]. These thresholds were selected based on their established validity for detecting early carious lesions on smooth surfaces.

### Data collection

#### Clinical parameters

Several clinical parameters were documented at baseline to characterize the sample and ensure group comparability. These included plaque index scores to assess oral hygiene status, gingival index measurements to evaluate periodontal health, decayed-missing-filled teeth (DMFT) scores to quantify caries experience, and the duration of clear aligner treatment to account for exposure time. All clinical parameters were collected by calibrated examiners using standardized protocols.

#### Behavioral factors

Participant behavioral factors related to oral hygiene and dietary habits were assessed through structured questionnaires. These included daily tooth brushing frequency, flossing habits, frequency of sugary food and beverage consumption, and adherence to professional oral hygiene recommendations. Behavioral data were collected to identify potential confounding factors that might influence demineralization outcomes independent of the assigned remineralization protocol.

Compliance with the assigned home-applied remineralization protocols (Groups B and C) was assessed using patient self-report diaries. Participants recorded the daily application of their assigned agents. These diaries were reviewed by the examiner at each monthly follow-up visit, and compliance was calculated as (total days applied/total days in the period) × 100. Compliance for Group D was considered 100%, as the agent was professionally applied at all scheduled follow-up visits.

### Statistical analysis

As multiple teeth were obtained from each participant and repeated measurements were taken from each tooth at four different time points, the data had a hierarchical and repeated-measures structure. Therefore, a linear mixed-effects model (LMM) was used to account for within-subject and within-tooth correlations [19]. In the model, time was treated as a repeated factor and the teeth were specified as nested within the subjects. Random intercepts were included for both the subjects and teeth to allow for individual variability at each level. Fixed effects included group, time, age, gender and number of attachments. A diagonal covariance structure was applied to model the correlations among repeated observations over time. The model parameters were estimated using the Restricted Maximum Likelihood (REML) method. The significance of the fixed effects was assessed using Type III F-tests. When a significant interaction was detected, the estimated marginal means (EMMEANS) and pairwise comparisons were used to examine differences between groups across time points. A p-value < 0.05 was considered statistically significant.

Post-hoc power analysis was performed using G*Power 3.1 [20] to evaluate the study’s power given the observed effect size (partial η² = 0.974).

During the preparation of this manuscript, the authors used AI language models (Google Gemini and ChatGPT) for language enhancement (grammar, syntax, and clarity) and formatting assistance. The authors have reviewed and edited the output and take full responsibility for the content of this publication.

## Results

### Participant demographics

**Fig. 1:**
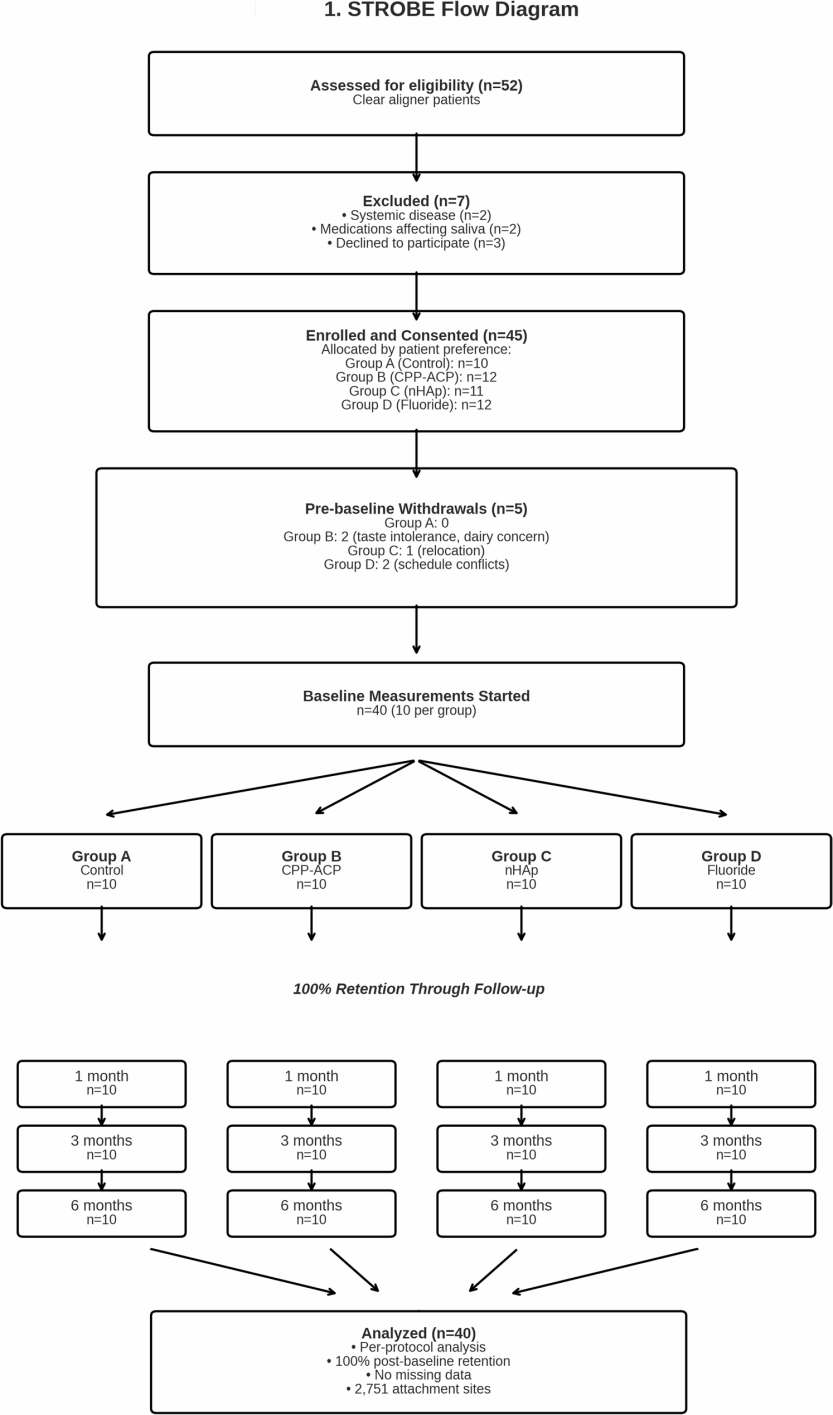
STROBE flow diagram of participant enrollment, allocation, and follow-up. The study assessed 52 patients for eligibility, excluded 7 based on predefined criteria, and enrolled 45 participants. Following allocation by patient preference and 5 pre-baseline withdrawals, 40 participants (10 per group) initiated baseline measurements. All participants completed the 6-month follow-up (100% post-baseline retention) and were included in the per-protocol analysis. A total of 2,751 attachment sites were measured

Of the 52 patients screened, 7 were excluded and 45 consented to participate. Five pre-baseline withdrawals occurred: two from the CPP-ACP group (product taste intolerance with nausea and dairy allergy concerns), one from the nHAp group (city relocation), and two from the fluoride varnish group (both citing the inability to attend regular clinical appointments). The control group had no withdrawal. The final cohort of 40 patients who initiated baseline measurements demonstrated 100% retention at the 6-month follow-up. This per-protocol population comprised 60% females, mean age 22.9 ± 7.0 years (range: 16–45). Dropout rates were not significantly different between the initial allocation groups (Fisher’s Exact Test, *p* = 0.382).(Fig. 1).

### Missing data

All 40 participants who initiated the study protocol completed the 6-month follow-up, resulting in a 0% post-baseline dropout rate. Therefore, no participant-level missing-data imputation was required for the primary analysis population (*n* = 40).

Site-level exclusions (i.e., DIAGNOdent measurements not obtained on teeth without attachments or on absent teeth) were by design and did not represent missing data requiring imputation. Of the theoretical maximum of 4480 potential measurement sites (40 patients × 28 teeth × 4 time points), 2751 (61.4%) corresponded to existing teeth with attachments and were included in the site-level dataset analysis informing the LMM.

The primary outcome was the change in DIAGNOdent scores from baseline (T0) to 6 months (T3). Secondary outcomes included the comparison of treatment efficacy among the three active interventions and assessment of temporal patterns in demineralization changes across measurement time points.

### Baseline characteristics (Table 1)

**Table 1:** Demographics and baseline characteristics of study participants

Characteristic	Group A (Control)	Group B (CPP-ACP)	Group C (nHAp)	Group D (Fluoride)	Total	*p*-value
n	10	10	10	10	40	-
Age (years), Mean ± SD	22.6 ± 6.7	22.5 ± 6.0	23.8 ± 8.5	22.7 ± 6.6	22.9 ± 7.0	0.92†
Range	16–38	16–35	16–45	16–36	16–45	
Sex, n (%) Female	6 (60.0)	5 (50.0)	6 (60.0)	7 (70.0)	24 (60.0)	0.68‡
Male	4 (40.0)	5 (50.0)	4 (40.0)	3 (30.0)	16 (40.0)	
Baseline DIAGNOdent, Mean ± SD	10.4 ± 2.5	12.3 ± 1.5	12.1 ± 0.7	12.1 ± 0.9	11.5 ± 1.6	0.48†
Median (IQR)	9.5 (7.0–12.0)	11.0 (9.0–15.0)	11.0 (8.0–15.0)	11.0 (8.5–14.5)	10.5 (8.0–14.0)	
DIAGNOdent Category, n (%) Sound (0–12)	7 (70.0)	5 (50.0)	5 (50.0)	5 (50.0)	22 (55.0)	0.76‡
Initial (13–24)	3 (30.0)	4 (40.0)	4 (40.0)	4 (40.0)	15 (37.5)	
Advanced (≥ 25)	0 (0.0)	1 (10.0)	1 (10.0)	1 (10.0)	3 (7.5)	
**Initial enrollment (n)**	10	12	11	12	–	–
**Dropout rate (%)¹**	0%	16.7%	9.1%	16.6%		0.382²

**Fig. 2:**
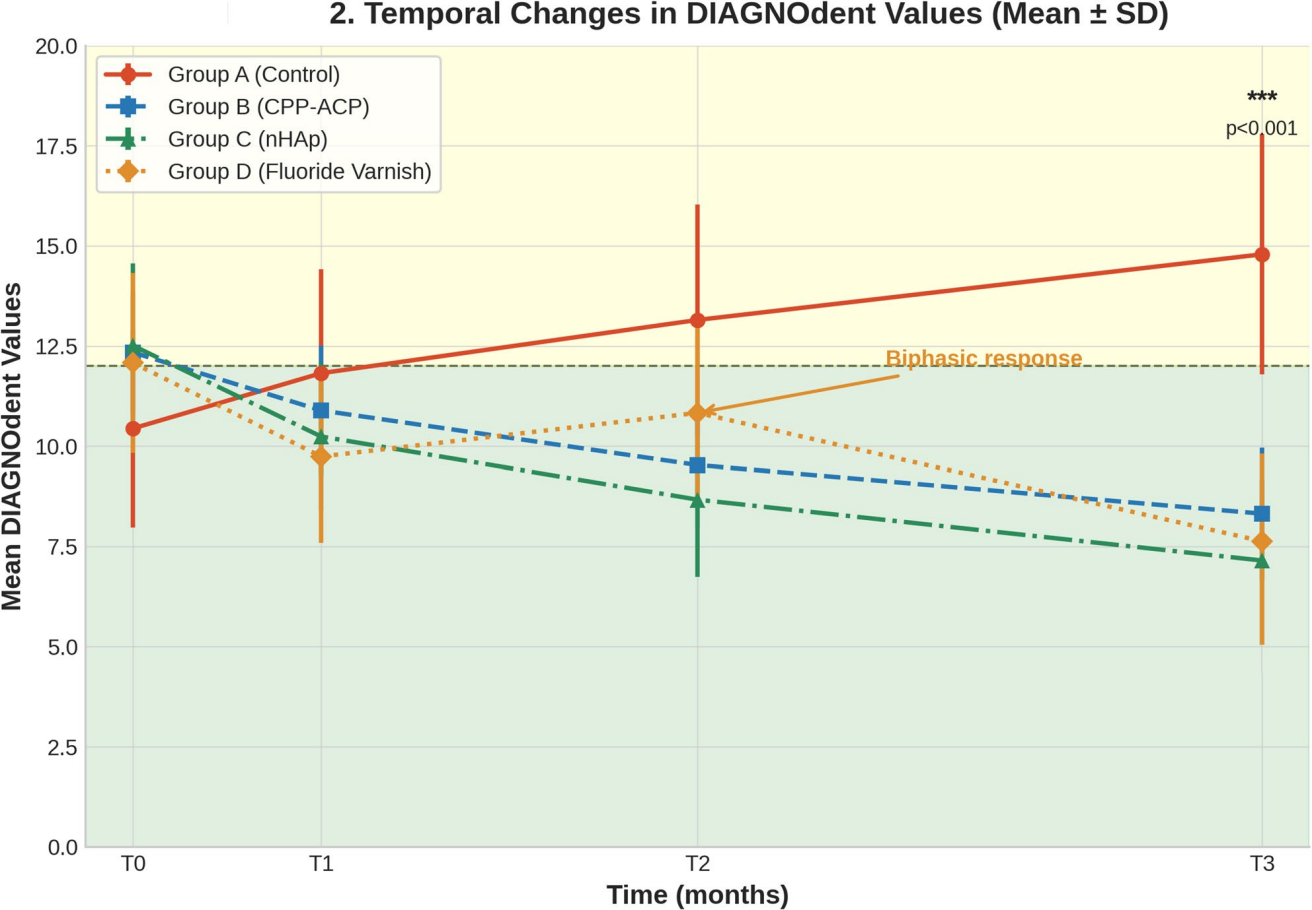
Temporal changes in DIAGNOdent values (mean ± SD) across four treatment groups over 6 months. Shaded areas represent clinical thresholds: green (0-12) indicates sound enamel, yellow (12-24) indicates initial demineralization. The dashed line at 12 marks the transition threshold. Note the biphasic response pattern in the fluoride varnish group at 3 months. Error bars represent standard deviation; n=10 per group

At baseline (T0), mean DIAGNOdent values ranged from 10.44 ± 2.46 (Group A) to 12.34 ± 1.48 (Group B). One-way ANOVA revealed no significant between-group differences in baseline DIAGNOdent scores (F(3,36) = 1.85, *p* = 0.48), confirming a comparable initial enamel status across all groups (Table 1). The mean number of attachments differed significantly between groups, with Group C having more attachments (18.9 ± 2.4) than the other groups (16.1–17.3; *p* = 0.007). Table 2 shows all timepoint values, and Fig. 2 depicts the temporal trends.

**Table 2:** DIAGNOdent values at each timepoint by treatment group showing Temporal changes from baseline (T0) to 6 months (T3)

Group	Baseline (T0) Mean ± SD [95% CI]	1 Month (T1) Mean ± SD [95% CI]	3 Months (T2) Mean ± SD [95% CI]	6 Months (T3) Mean ± SD [95% CI]	Δ (T3 − T0) Mean [95% CI]	*p*-value †
**Control (A)**	10.4 ± 2.5 [8.7–12.2]	11.5 ± 2.5 [9.7–13.2]	12.2 ± 2.6 [10.3–14.1]	14.8 ± 2.6 [13.0–16.6]	+ 4.35 [3.77–4.93]	< 0.001
**CPP-ACP (B)**	12.3 ± 1.5 [11.3–13.4]	11.1 ± 1.6 [10.0–12.3]	9.9 ± 1.3 [9.0–10.8]	8.3 ± 1.2 [7.4–9.2]	−4.02 [− 4.47 to − 3.56]	< 0.001
**nHAp (C)**	12.1 ± 0.7 [11.6–12.6]	10.0 ± 0.9 [9.3–10.6]	8.8 ± 0.7 [8.2–9.3]	6.7 ± 0.6 [6.2–7.1]	−5.36 [− 5.99 to − 4.72]	< 0.001
**Fluoride (D)**	12.1 ± 0.9 [11.5–12.7]	9.7 ± 1.1 [9.0–10.5]	10.8 ± 1.0 [10.1–11.6]	7.6 ± 0.8 [7.0–8.1]	−4.45 [− 4.78 to − 4.11]	< 0.001

### Primary outcome analysis

**Fig. 3:**
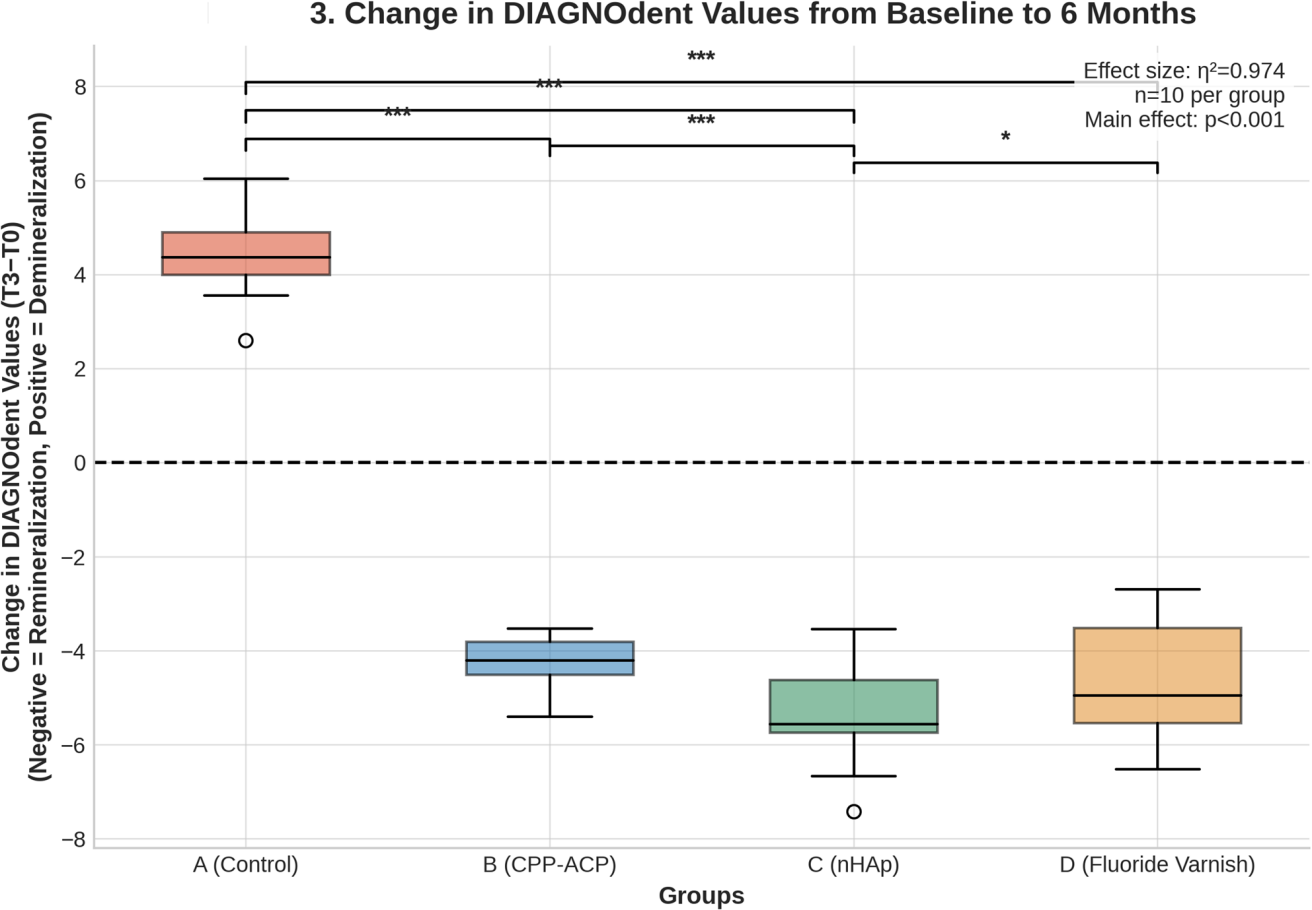
Box plots showing change in DIAGNOdent values from baseline to 6 months (T3-T0). Negative values indicate remineralization; positive values indicate demineralization. The dashed line at y = 0 represents no change. Boxes show IQR with median line; whiskers extend to min/max values; circles indicate outliers. Significance: ***p < 0.001, *p < 0.05. The nHAp group demonstrated the greatest reduction, significantly outperforming both CPP-ACP (*p* < 0.001) and fluoride varnish (*p* = 0.019)

The primary analysis using a linear mixed-effects model (LMM), adjusting for baseline attachment number as a covariate, revealed a highly significant main effect of GROUP on the change in DIAGNOdent scores from baseline to 6 months (F [3, 35] = 438.4, *p* < 0.001). The effect size was exceptionally large (partial η² = 0.974). The covariate baseline attachment number was not significantly associated with the outcome (*p* = 0.470). The distribution of these changes is illustrated in Fig. 3.

### Pairwise comparisons

Post-hoc pairwise comparisons with Bonferroni correction confirmed that all active treatment groups (B, C, and D) showed significantly greater reduction in DIAGNOdent scores than the control group (A) (*p* < 0.001 for all). In contrast, the control group (Group A) showed significant worsening with a mean increase of + 4.35 in DIAGNOdent scores (*p* < 0.001), confirming the risk of attachment-associated demineralization without preventive intervention. Crucially, the nHAp group (C) demonstrated a significantly greater reduction compared to both the CPP-ACP group (B) (Mean Difference = −1.34 units, *p* < 0.001) and the fluoride group (D) (Mean Difference = −0.91 units, *p* = 0.019). No significant difference was found between the CPP-ACP (B) and fluoride (D) groups (Mean Difference = −0.43 units, *p* = 0.375).

### Temporal patterns

**Fig. 4:**
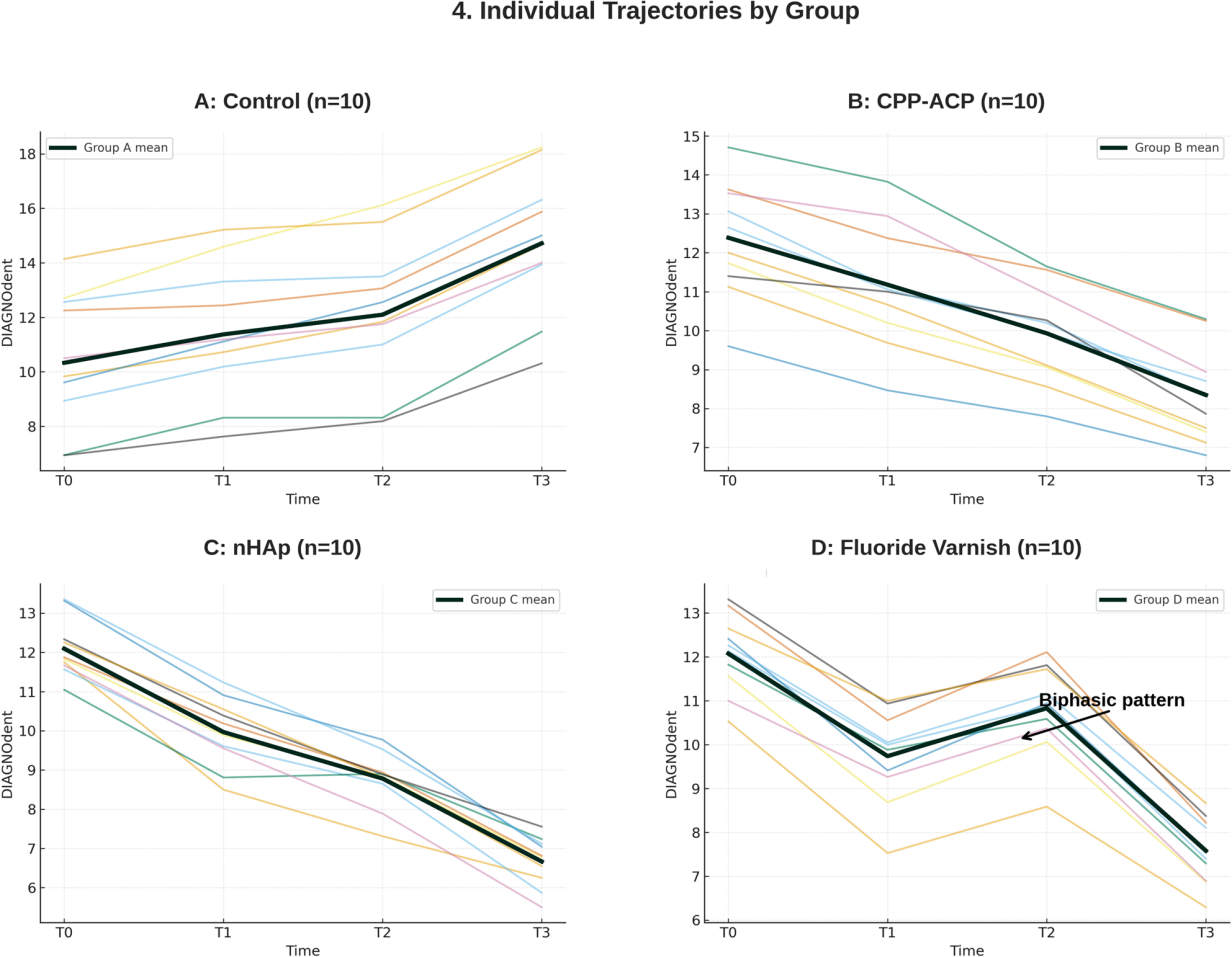
Individual patient trajectories (spaghetti plots) showing DIAGNOdent values over 6 months for all study participants. Each thin colored line represents an individual patient (*n* = 10 per group); thick dark lines indicate group means. **A** Control group shows consistent deterioration across all patients. **B** CPP-ACP group demonstrates uniform improvement. **C** nHAp group exhibits the greatest and most consistent reduction. **D** Fluoride varnish group displays characteristic biphasic response pattern with temporary worsening at 3 months in most patients before final improvement

Analysis of temporal patterns revealed distinct trajectories across the measurement intervals. Groups B (CPP-ACP) and C (nHAP) demonstrated progressive, monotonic reductions at each timepoint, indicating consistent remineralization throughout the study period. Group D (Fluoride) exhibited a biphasic response pattern with initial reduction from baseline to T1 (12.08→9.74), followed by a transient increase from T1 to T2 (9.74→10.83), and subsequent final reduction from T2 to T3 (10.83→7.63), suggesting an initial adjustment phase before sustained remineralization. In contrast, Group A (Control) showed progressive increases in DIAGNOdent scores throughout the study period, confirming continuous demineralization without intervention. The individual patient trajectories are shown in Fig. 4.

### Subgroup analyses

#### Age-based analysis

The age cutoff of 18 years was selected to differentiate between adolescent patients, who are typically in the active growth phase and may have different compliance levels compared to adults [4, 21]. Additionally, recent studies have specifically highlighted unique risk factors for white spot lesions in adolescent patients undergoing clear aligner therapy [5]. Age-based subgroups (< 18 vs. ≥18 years) showed similar treatment responses (p(interaction) = 0.84). All agents were effective regardless of the age category.

#### Tooth-type analysis

Posterior teeth exhibited a slightly higher mean recovery than anterior teeth (−6.1 vs. −4.6 units, *p* = 0.02), which was most pronounced in the nHAp and fluoride groups.

### Adverse events

No adverse effects, including tooth hypersensitivity, gingival irritation, or enamel discoloration, were reported during the 6-month observation period. Self-reported compliance for home-applied agents (Groups B and C) exceeded 95% based on diary reviews. All participants in Group D completed their scheduled professional applications. All remineralization protocols were well tolerated.

## Discussion

This prospective controlled clinical trial rejected the null hypothesis, demonstrating significant differences among remineralization protocols in preventing attachment-associated demineralization during clear aligner therapy (*p* < 0.001, η²*p* = 0.974).

This study provides the first quantitative assessment of remineralization agent effectiveness, specifically around clear aligner attachments, addressing a critical gap in the orthodontic literature. Our findings demonstrate that all three remineralization protocols—nHAp, fluoride varnish, and CPP-ACP—significantly reduced enamel demineralization compared to standard fluoride toothpaste alone, with nHAp showing the greatest effect (5.36 unit reduction in DIAGNOdent values).

The 42% increase in DIAGNOdent values observed in the control group over 6 months aligns with Liu et al.‘s finding that anterior attachments represent an independent risk factor for WSL development (OR = 2.192) [5]. This deterioration, despite standard fluoride toothpaste use, underscores the inadequacy of conventional oral hygiene alone in preventing attachment-associated demineralization. The attachment-enamel interface creates a unique microenvironment that facilitates biofilm accumulation and acid retention, explaining the progressive demineralization observed [5].

Our finding that nHAp demonstrated superior efficacy (−5.36 units) compared to both CPP-ACP (−4.02 units) and fluoride varnish (−4.45 units) contrasts with El Mansy et al.‘s hierarchy of post-orthodontic WSLs, where the difference between agents was less pronounced [15]. This discrepancy may reflect the specific challenges of the attachment environment, where the biomimetic properties of nHAp and its ability to directly integrate with the enamel structure provide particular advantages. Nano-sized particles (20–80 nm) can penetrate the microscopic irregularities at the attachment-enamel interface more effectively [22].

The biphasic response pattern observed in the fluoride varnish group—initial improvement (T0→T1), transient worsening (T1→T2), and final improvement (T2→T3)—aligns with the established fluoride release kinetics. Recent systematic reviews have confirmed that fluoride release from varnishes typically follows a biphasic pattern that begins with a short-term peak in fluoride availability, after which levels gradually decrease [23]. This fluctuation likely reflects the specific release kinetics of fluoride varnishes, characterized by an initial ‘burst effect’ of high ion release followed by a rapid decline [24]. Unlike smooth surfaces, the irregular topography of attachment margins may compromise the retention of the varnish layer, causing the fluoride reservoir to deplete before the 3-month mark. The re-application of varnish at the 3-month visit (T2) likely replenished this reservoir, explaining the subsequent improvement observed at the 6-month endpoint [25]. This suggests that quarterly applications may require more frequent applications for high-risk attachment environments.

The consistent progressive improvement observed with daily home-applied agents (nHAp and CPP-ACP) supports the importance of continuous remineralization therapy. This effect was likely enhanced by the methodology of using the aligners themselves as delivery trays. This specific mode of application—using aligners as carriers—ensures prolonged contact time at the peri-attachment site, a finding supported by a recent RCT that also utilized tray-based delivery of an F-ACP mousse to successfully manage post-orthodontic WSLs [7]. Unlike episodic high-concentration exposure from professional varnish, these agents maintain steady-state mineral supersaturation in the peri-attachment environment. The superior performance of nano-hydroxyapatite may be attributed to its dual remineralization mechanism, functioning both as a source of calcium and phosphate ions and as a template that promotes epitaxial crystal growth on demineralized enamel surfaces [26, 27].

Our results demonstrated greater effect sizes (η²=0.974) than those typically reported in fixed appliance studies, wherein fluoride varnish reduces WSL incidence by approximately 36% [28]. This difference may reflect several factors. First, the removable nature of aligners allows better agent delivery during tray wear, as patients can apply remineralization agents directly to tooth surfaces before aligner insertion. Second, attachment surfaces are more accessible than bracket-adjacent enamel, facilitating more thorough cleaning and agent application. Third, our quantitative DIAGNOdent monitoring may detect subtle changes missed by visual assessment methods commonly used in fixed appliance studies, potentially capturing early remineralization effects that would otherwise go unnoticed.

The 100% post-baseline retention rate in our study, while remarkable, likely reflects the motivated patient population seeking clear aligner therapy and the noninvasive nature of the interventions. Notably, all five withdrawals occurred before baseline measurements, primarily because of product intolerance or scheduling conflicts, emphasizing the importance of trial applications and thorough informed consent processes. This contrasts with fixed-appliance cohorts, where discontinuation rates are typically around 8–10% in NHS datasets, with ~ 4–15% reported across European studies (occasionally up to ~ 20% depending on the setting) [29–31].

The DIAGNOdent cut-off values used (0–12: sound, 13–24: initial demineralization, ≥ 25: advanced) align with Kim et al.‘s validated thresholds for smooth surfaces [18, 32]. The mean baseline values (10.4–12.3) indicated that most patients began with sound enamel or borderline demineralization, suggesting that attachment placement itself may initiate subclinical changes detectable only through quantitative methods.

The economic implications merit further consideration. Professionally applied fluoride varnish, typically delivered every 3–6 months per ADA recommendations, costs approximately US $20–50 per application [33]. In contrast, CPP-ACP creams (e.g., GC Tooth Mousse/MI Paste) and n-HAp toothpastes (e.g., Boka, RiseWell) represent modest home-care expenses, averaging US $10–30 per month [34]. Preventing even a single cavitated lesion can offset a restorative expense averaging ≈ US $200 per tooth, highlighting the preventive value of remineralization protocols [35].

Several limitations of this study warrant discussion. First, the non-randomized allocation based on patient preference introduces potential selection bias, although the baseline characteristics were well balanced between the groups. Patients who chose active interventions may have been more motivated to maintain oral hygiene. Second, Second, the primary limitation of this study is the small convenience sample (*n* = 10 per group) without formal a priori sample size calculation. Conventional power analysis using standard effect sizes (Cohen’s f = 0.40) would have required approximately 19 participants per group for 80% power, suggesting that our study was initially underpowered [36]. However, post-hoc power analysis using G*Power 3.1 [20] revealed that the exceptionally large effect size observed (partial η² = 0.974) yielded statistical power exceeding 0.99, indicating that the study was adequately powered to detect substantial treatment differences despite the small sample size per group. Nevertheless, this exploratory finding requires confirmation in larger, adequately powered trials with pre-specified sample size calculations. Although the high effect size compensates for the small sample size statistically, the generalizability of these findings to broader populations with different oral hygiene habits, socioeconomic backgrounds, or geographic regions remains to be confirmed in larger multi-center trials. The exploratory nature of this pilot study necessitates cautious interpretation. Third, the single-center design of a university setting may not reflect community practice patterns.

The lack of blinding, inherent to the nature of the intervention, could introduce performance and detection bias. However, DIAGNOdent provides objective quantitative measurements that are less susceptible to examiner bias than are visual scoring systems. The single-examiner protocol, while ensuring consistency (ICC = 0.85), limits the external validity assessment. While the normality assumption for the residuals was generally not met, the robustness of the LMM to such deviations, combined with the confirmation of variance homogeneity (Levene’s test, *p* = 0.896), supports the validity of the analysis.

Although a significant difference in baseline attachment numbers (*p* = 0.007) was noted between the groups, our primary linear mixed-effects model analysis was adjusted for this variable as a covariate. The analysis confirmed that baseline attachment number did not significantly influence the primary outcome (*p* = 0.470), indicating that the observed group differences were robust to this initial imbalance. Additionally, Group C received both professional in-office nHAp applications (ProOffice gel at T0, T1, T2) and daily home-applied nHAp gel (Nanocare), representing a more intensive protocol than the other groups. These factors should be considered when interpreting Group C’s superior outcomes. Future studies should stratify randomization by attachment number and standardize dosing protocols.

Strengths include the prospective design, complete follow-up, validated measurement protocol, and focus on a clinically relevant, but understudied population. The 6-month duration captures the medium-term effects relevant to typical clear aligner treatment phases. The use of commercially available products has enhanced their immediate clinical applicability.

Our findings raise several research questions: First, would more frequent fluoride varnish application (monthly or bimonthly) eliminate the biphasic pattern? Second, would combining agents (e.g., daily nHAp plus quarterly fluoride varnish) provide synergistic effects? Third, can baseline DIAGNOdent values predict individual responses to specific agents enabling personalized prevention protocols?

Long-term studies should assess whether early intervention prevents cavitation that requires restorative treatment. Microbiological assessments could elucidate whether remineralization agents also modify the composition and metabolic activity of cariogenic biofilms [37, 38].

Regarding clinical significance, the reduction of approximately 5 DIAGNOdent units observed in the nHAp group represents a meaningful shift in enamel quality. A strong positive correlation (*r* = 0.892) between DIAGNOdent readings and ICDAS-II scores has been demonstrated for smooth surface lesions [39]. According to validated thresholds where values < 13 indicate sound enamel, the observed reduction from baseline (12.1) to final (6.7) signifies a decisive transition from borderline demineralization back to the ‘sound’ category. This quantitative improvement corresponds to a clinically visible arrest of white spot lesion progression, equating to a shift from ICDAS code 1–2 (initial lesion) to ICDAS 0 (sound surface) [39, 40]. Economic analyses comparing preventive agent costs against potential restoration expenses would inform healthcare policies.

## Conclusions

This prospective study provides critical evidence highlighting the significant iatrogenic risk associated with untreated clear aligner attachment sites, demonstrating a substantial increase in demineralization over 6 months despite standard fluoride toothpaste use. However, our findings offer clear solutions; targeted active remineralization protocols can effectively mitigate this complication. Among the tested regimens, the intensive nano-hydroxyapatite protocol (5% professional application plus 1% daily home use) demonstrated superior efficacy, yielding a covariate-adjusted reduction of 5.36 units, which was significantly greater than that of both CPP-ACP and quarterly fluoride varnish. Although the fluctuation observed in the quarterly fluoride varnish group requires further study, all active protocols significantly outperformed the control.

These findings challenge the paradigm of passive monitoring, which is often employed in clear aligner therapy. Rather than accepting WSLs as an inevitable side effect, our data strongly support the implementation of active prevention strategies from the initiation of treatment, particularly in patients with multiple attachments. The observed DIAGNOdent reductions, particularly with the nHAp protocol, likely correspond to the shifting enamel status from ‘initial demineralization’ towards ‘sound enamel’ classifications, underscoring the clinical relevance of early intervention. For immediate clinical application, practitioners should consider risk stratification (e.g., attachment number) and patient compliance potential when selecting a remineralization protocol utilizing quantitative monitoring such as DIAGNOdent to guide interventions.

While the exploratory nature, non-randomized design, and small sample size of this pilot study necessitate cautious interpretation, the remarkably large effect size (partial η² = 0.974) and 100% post-baseline retention rate provided compelling preliminary evidence. This study represents the first step towards personalized preventive orthodontics, where risk assessment, real-time monitoring, and targeted interventions can potentially eliminate iatrogenic WSLs. Larger, adequately powered randomized controlled trials with extended follow-up are warranted to confirm these findings, establish the minimal clinically important difference (MCID, the smallest change considered clinically meaningful) for DIAGNOdent changes, and update clinical guidelines for preventing attachment-associated enamel demineralization in the rapidly growing clear aligner patient population.

## Supplementary Information

Supplementary Material 1.

## Data Availability

The datasets generated and analyzed during the current study are available from the corresponding author, Banu Kılıç (bkilic@bezmialem.edu.tr), on reasonable request.

## Abbreviations

AIC: Akaike Information Criterion
ANOVA: Analysis of variance
BIC: Bayesian Information Criterion
CI: Confidence interval
CPP-ACP: Casein phosphopeptide-amorphous calcium phosphate
ICC: Intraclass correlation coefficient
LMM: Linear mixed-effects model
MCID: Minimal clinically important difference
nHAp: Nano-hydroxyapatite
OR: Odds ratio
REML: Restricted maximum likelihood
SD: Standard deviation
T0: Baseline (initial measurement)
T1: First follow-up (1 month)
T2: Second follow-up (3 months)
T3: Third follow-up (6 months)
WSL: White spot lesions
