# Supplementary figures and images for: Comparative effectiveness of remineralization agents on attachment-associated enamel demineralization in clear aligner patients: A 6-Month DIAGNOdent-Based controlled clinical trial
Source: BMC Oral Health. 2025 Dec 11;26:96. doi: 10.1186/s12903-025-07497-8 (PMC12801708; doi:10.1186/s12903-025-07497-8)

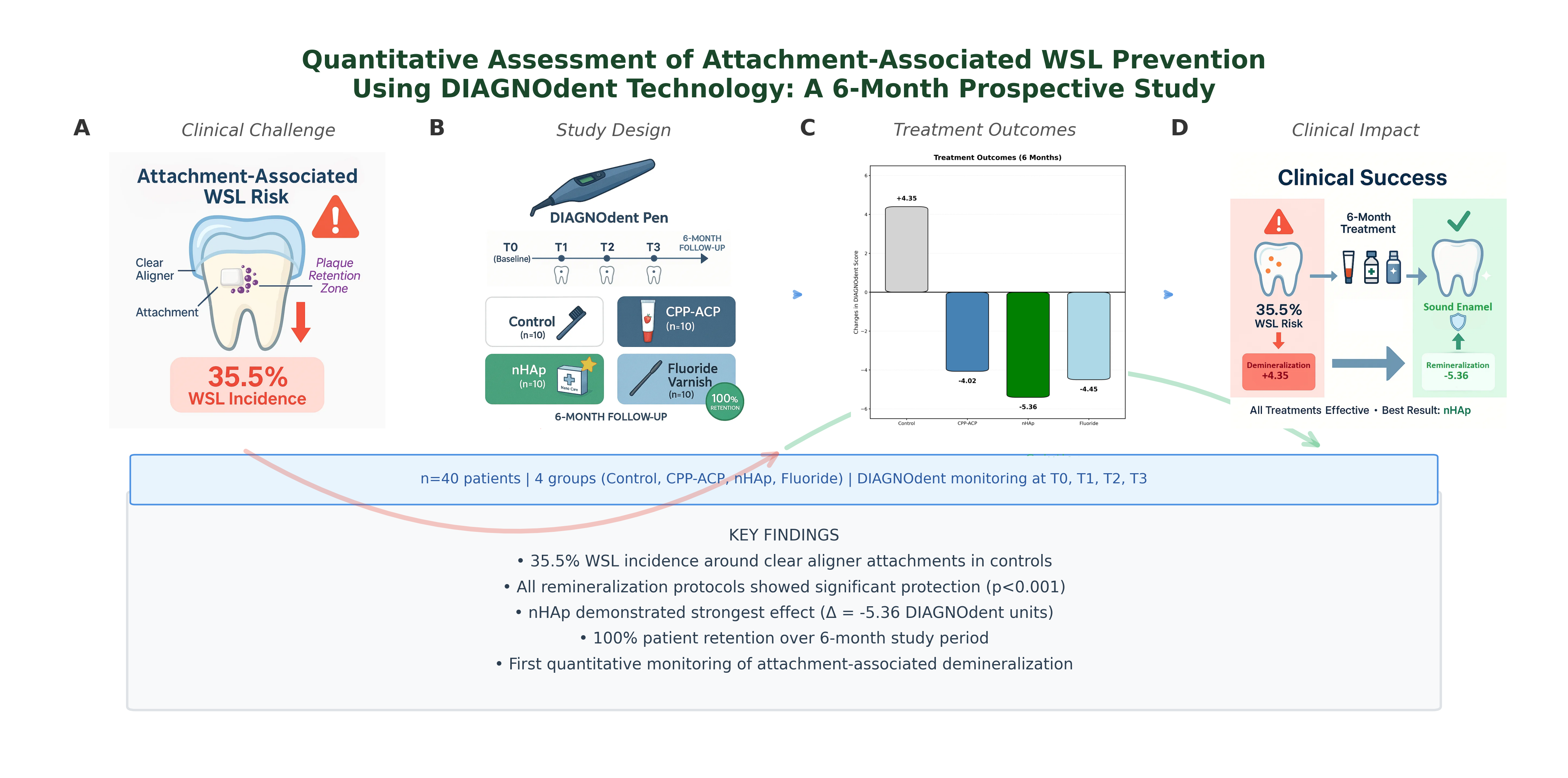

Supplement: Supplementary file 1 — Supplementary Material 1. [file 12903_2025_7497_MOESM1_ESM.png]
